# Implementing interprofessional learning curriculum: how problems might also be answers

**DOI:** 10.1186/s12909-018-1231-1

**Published:** 2018-06-08

**Authors:** Maree O’Keefe, Helena Ward

**Affiliations:** Faculty of Health and Medical Sciences, The University of Adelaide, Adelaide, Australia

**Keywords:** Interprofessional learning, Health, Curriculum, Activity theory

## Abstract

**Background:**

Despite interprofessional learning (IPL) being widely recognised as important for health care professions, embedding IPL within core curriculum remains a significant challenge. The aim of this study was to identify tensions associated with implementing IPL curriculum for educators and clinical supervisors, and to examine these findings from the perspective of activity theory and the expansive learning cycle (ELC).

**Methods:**

We interviewed 12 faculty staff and ten health practitioners regarding IPL. Interviews were semi-structured. Following initial thematic analysis, further analysis was undertaken to characterise existing activity systems and the contradictions associated with implementing IPL. These findings were then mapped to the ELC.

**Results:**

Five clusters of contradictions were identified: the lack of a workable definition; when and what is best for students; the leadership hot potato; big expectations of IPL; and, resisting cultural change. When mapped to the ELC, it was apparent that although experienced as challenges, these contradictions had not yet generated sufficient tension to trigger ‘break through’ novel thinking, or contemplation and modelling of new solutions.

**Conclusions:**

The application of activity theory and the ELC offered an approach in which the most troublesome challenges might be reframed as opportunities for change. Seemingly intractable problems could be worked on to identify and address underlying fears and assumptions. If sufficient tension can be generated, an ELC could then be triggered. In reframing challenges as opportunities, the power of tensions and contradictions as potential levers for effective change might be more successfully accessed.

## Background

It is no longer appropriate that future health care professionals be trained solely within their respective disciplines. Interprofessional learning (IPL) has developed in response to a need for students of different disciplines to learn ‘with, from and about each other’ [1, 2]. Despite IPL being widely described as important, and the existence of an internationally endorsed definition, embedding IPL into standard health profession curriculum remains a significant challenge [3–8]. Commonly cited reasons for this situation include logistic and cultural factors such as timetabling constraints, cost, risk-averse departmental cultures and the specific requirements set by individual disciplines and/or professional bodies [9–16]. More recently assumptions that health professional roles are stable and defined have been challenged adding further complexity to IPL curriculum development and delivery [17]. Reporting of IPL activities in the literature also varies greatly with calls for greater consistency in reporting model development and testing [18].

In this paper we describe experiences at one university where an IPL curriculum was being developed and implemented. Being aware of the published literature around the frequently encountered challenges and barriers to successful IPL initiatives, we sought to understand these potential impediments within our own environment with the intent of identifying and promoting local solutions early in the process.

One of the major challenges faced when designing and implementing IPL is the tension between competing interests. Curriculum designed to deliver IPL competencies should not be confused with curriculum designed to deliver core disciplinary competencies using IPL teaching approaches [5]. Where these two distinct curriculum intentions become intertwined, students and teachers may perceive, either implicitly or explicitly, that competing agendas between disciplinary and IPL activities are in play. If this happens disciplinary imperatives will usually take precedence with individual faculty academics focusing predominantly on their own profession [8, 19]. In the specific context of clinical placements, a similar situation may be expressed as tension between discipline specific patient management and the push for collaborative patient care, with collaboration being sacrificed when disciplinary autonomy is threatened [20]. Similarly, if tension exist around perceived authority, trust and respect arising from real or perceived power differentials between disciplines, IPL may become a contested space within the curriculum [19, 20]. Too often the solution to managing these tensions is to ‘bolt-on’ IPL learning activities rather than integrating them more securely into the core curriculum [1, 5, 6, 21].

In addition to the commonly cited challenges to implementing IPL described above, understandings of what an optimal IPL curriculum might comprise are also evolving. The extent to which IPL activities focus on competencies (specific knowledge and skills) and/or capability development (ability to deal with change and uncertainty) can vary. Whereas competencies measure performance of a task, curriculum that develops capability may be conceived as extending learning to build individuals’ ability to adapt and generate new ideas to improve performance [22]. It has been suggested that ‘interprofessionality’ itself may be a core capability linking IPL and subsequent interprofessional collaborative practice [23–25].

Socio-cultural learning theories such as activity theory and expansive learning offer a fruitful avenue for understanding how various factors might be contributing to or hindering more IPL integration into health profession curriculum [26–28]. Activity theory describes a perspective in which goal-oriented activity, such as patient care or student clinical supervision, is conceived as consisting of a number of different activity systems. Each of these activity systems includes the person or team (the subject), the object or purpose of the work and the tools or enablers that are available to transform the object into an outcome. There is a focus on the various elements that contribute to change and innovation in a system with learning occurring at an organisational or system level [28–30].

One of the strengths of activity theory as a lens to explore IPL, is that it takes account of the inherent instability and fluidity of interprofessional activity. This activity may involve various groupings of health care professions with complementary scopes of practice and approaches to patient care coming together for different clinical interactions. With a focus on change and the dynamics of learning, the unit of analysis is joint human activity or practice embedded within a social context. For example, in a hospital ward, a pharmacist (the subject) may be teaching students from different health professions about the importance of correct drug prescribing and confirming patient information (the object of the activity). The learning activity may occur by way of patient interaction and/or review of drug charts and patient case notes (tools). The outcome of this activity system may be reduction in future prescribing errors. Within such an activity system, tensions will arise if the object is difficult to achieve due to perhaps appropriate tools being unavailable (in this case, unavailability of the patient case notes and/or drug charts) or the patient is unavailable. In either case the student learning activity cannot be completed as intended, potentially compromising the outcome of reduced prescribing errors in the future. These tensions, or contradictions, will appear as barriers to successful learning in this instance.

In more complex environments, many interrelated activity systems will co-exist and contradictions may arise between different activity systems. In the case of IPL, the need to engage students in IPL activities (one object) when there is a responsibility to ensure discipline specific learning outcomes are achieved (a different object) may be understood as a contradiction. It is often the case that challenges are viewed as barriers to successful implementation of IPL. An alternate perspective is that each of these barriers could be understood as a legitimate contradiction. That is, two activity systems (in this example disciplinary learning activities and IPL activities) that are in tension. If sufficient tension is generated, an expansive learning cycle (ELC) can be triggered.

The ELC is described as a staged sequence of learning actions. When fully realised, tensions associated with contradictions become sufficiently intense that they trigger breakthrough thinking with new solutions being developed and modelled that result in a transformed object of the activity (Table 1). In such instances, tensions and contradictions can be viewed, not as barriers, but as ‘forces of change and development’ [31].

**Table 1:** Sequence of learning actions associated with expansive learning cycle [28]

1. Questioning/Need	
2. Analysis/Double bind	
3. Modeling the new solution/Breakthrough	
4. Examining and testing the new model/Adjustment, enrichment	
5. Implementing the new model/Resistance	
6. Reflecting on the process/Stabilisation	
7. Consolidating and generalizing the new practice	

Where there is tension between, for example, IPL and discipline teaching, the transformed object might be one where IPL and disciplinary learning occur together. As joint, not individual activity, is the unit of analysis, if there is general agreement to such a curriculum change, developing and implementing this new solution will proceed thus completing the cycle [28, 32].

In previous work implementing change in the management of student clinical placements, the theoretical perspective of activity theory and the associated ELC provided useful insight into why some initiatives succeeded while others did not [32, 33]. The aim of this study was to identify tensions associated with implementing IPL curriculum for educators and clinical supervisors, and to examine these findings from the perspective of activity theory and the ELC.

## Methods

This study was undertaken at the University of Adelaide in South Australia over an 18-month period in 2013–2015. At this time the Faculty of Health and Medical Sciences was about to move the schools of Dentistry, Medicine and Nursing from within the university to a new purpose-built university facility situated within a major health service precinct. The University offers undergraduate degree programs in dentistry, medicine and nursing. Prior to the move each school was housed in separate buildings dispersed across the main university campus. At the time of the study, the curriculum of each program was quite separate with very little shared teaching. Some small-scale pilot IPL activities had occurred. However, these were ‘one-off’ joint lectures or voluntary small group workshop or role play activities conducted outside usual timetabled teaching. The co-location of the three schools into one building within a health service precinct as opposed to a university campus presented an ideal opportunity to engage academic staff and clinical supervisors in the design and implementation of an embedded IPL curriculum that was shared by the three schools and health services more broadly. The scope of an IPL curriculum was conceived to be broad and to embrace classroom, simulation and/or clinical placement experiences. The University of Adelaide Human Ethics Committee approved the study and all participants provided written consent.

### Participants

Participants were identified through a purposive sampling matrix providing a mix of: health disciplines; university faculty staff and community health practitioners involved in teaching and/or supervising students; and, senior and junior staff. Selection of these criteria was informed by the practical need to ensure representation from the three schools and to include the perspectives of different groups of educators and clinical supervisors who would be engaged in delivering IPL. All invited participants agreed to be interviewed.

### Procedures

To facilitate participation, participants could choose either a face to face, video conference or telephone interview. Interviews were semi structured with question development by the authors informed by literature review and the aims of the study (Table 2). Questions were designed to invite open dialogue about IPL and were not informed by any specific theory.

**Table 2:** Interview guide

1. What is your understanding of interprofessional learning?	
2. What are the benefits of interprofessional learning?	
3. How should interprofessional learning be ‘taught’?	
4. What are the challenges with implementing interprofessional learning?	
5. What professional development might be needed for teachers?	

Interviews were audio recorded with participant consent. Audio files were subsequently transcribed and anonymised. We offered participants the opportunity to review their individual transcripts. The first author conducted 14 interviews. The second author conducted four interviews.

### Analysis

In the first round of analysis, transcripts were analysed using thematic qualitative techniques [34]. The authors read each transcript independently and undertook initial coding of content. Emerging themes were identified and matching of coded data within these themes occurred. We then compared our individual identified themes. Following iterative cycles of reviewing coded transcript data and refining themes, we then agreed on the final set of themes. All agreements were achieved by consensus and all coded data were captured within one of the identified themes. In allocating each piece of coded data, an easy and logical fit was required with the relevant theme. Where theme refinement was required this was undertaken by discussion with continued reference back to the original transcripts to maintain consistency and credibility of the analysis.

In the second round of analysis, we sought and characterised existing activity systems. With a particular focus on the activities and actions of educators and supervisors as described by the participants, we looked for specific tensions and contradictions that had emerged within or between these activity systems. Finally, we mapped our findings to the stages of the ELC.

At each stage of the analysis we reflected on our personal experiences in clinical practice, IPL curriculum development and delivery, and in educational research more generally and discussed how these experiences may have brought additional perspectives to the analysis. Particular care was taken during the analysis of transcripts where participants were known to us. This included ensuring anonymity which required additional measures to those usually employed such as limiting the description of participants rather than providing full details of the numbers sampled across each of the groups (discipline, university faculty or community health practitioner, and experience). Discussion following transcript review was strictly limited to the data with no referencing of the person. Participants were also assured of the rigorous observations of the requirement not to disclose any information provided in an interview that may be identifying.

## Results

Twelve faculty staff and ten community health practitioners participated in 18 interviews. Fifteen interviews occurred with a single participant, two with two participants (one faculty pair from medicine and one faculty pair from nursing) and one interview included three participants (a combination of medical and nursing faculty academics). Faculty participants included two dentists, six medical doctors, and four nurses. Community health practitioner participants included three medical doctors, three nurses, one occupational therapist, two speech pathologists and one pharmacist. Twelve interviews were conducted face to face, two interviews were conducted by videoconference, and four interviews were conducted by telephone. All interviews with more than one participant were conducted face to face. The duration of interviews ranged from 15 to 58 min (mean 26, median 24 min). The mean duration of face to face interviews was 31 min as compared with 16.5 min for telephone and 22 min for videoconference interviews. The duration of the two participant interviews was 26 and 44 min. The three participant interview was the longest at 58 min.

Participants described a variety of activity systems that shared the common object or purpose of delivering IPL to health profession students. Through our analysis we identified the following clusters of contradictions: the lack of a workable definition; when and what is best for students; the leadership hot potato; big expectations of IPL; and, resisting cultural change.

### The lack of a workable definition

Whether in the classroom or in clinical placements, participants understood there to be an apparently common object, that is to implement IPL within the curriculum. However, although there was some familiarity with the commonly cited definitions of IPL [1, 2], when encouraged to be more specific, participants found it very difficult to actually describe what they understood IPL to be, often resorting to examples of learning environments and describing aspects of clinical care rather than any particular theoretical, pedagogic or curriculum aspects. Where educational descriptions were offered, these tended to be less specific and more broadly based. There was frequent reference to the lack of a clear and shared understanding of IPL. Participant responses were often somewhat disorganised in content, with some participants openly acknowledging their confusion.

*there’s always a bit of vagueness between - even for me - intraprofessional, interprofessional, multidisciplinary. It all became - interdisciplinary. ……. So interdisciplinary or interprofessional? Now I’m getting confused.* (Community health practitioner 7)

*Well I think if there are difficulties defining [IPL] then it clearly means that we’re all talking different things and we haven’t got a clear view about what we want to achieve.* (Community health practitioner 3)

Several participants expressed the view that a flexible definition is good as it allows for an evolving meaning.

*I guess an advantage of having it ill-defined is that you can shape it to be whatever you want it to be in the context that you’re in, which has the flipside disadvantage of it’s hard to communicate what it is.* (Community health practitioner 10)

Tensions around the lack of a workable definition emerged as a clear impediment to progress for faculty staff who were frequently stuck on arguing definitions and unable to progress to discussion around implementation related activities. There was an understanding that IPL was to be implemented, yet faculty and community health practitioners could not articulate clearly what it was they were expected to implement.

### When and what is best for students

In addition to a need for clarity and specificity of definition for IPL to guide curriculum development and delivery, educators and clinical supervisors need access to effective and accessible pedagogical models. Participants expressed a range of opinions on the ways in which IPL could be achieved for students. As might be anticipated, faculty participants provided more comment on learning and teaching approaches than did community health practitioners. Across all the interviews though there was a focus on authentic learning experiences and a consensus that to be successful, IPL needed to be based around interactions between students.

*I don’t think it’s just sitting in a classroom together. I think there’s got to be some interaction between the students for it to be real, true interdisciplinary or IPL.* (Faculty 7)

IPL was considered a preferred approach to offering curriculum in more complex areas such as professionalism and ethics. The lack of a clear definition of IPL, or agreement on optimal teaching approaches though, led to confusion among many of the participants on what would be the best way to proceed in terms of implementation. Some faculty participants even questioned the extent to which IPL should be formally included in curriculum. They also sought to contain the scope of IPL whether for logistic reasons, for quality control, to ensure core disciplinary curriculum was delivered or just to make complexity manageable. These comments gave the impression that faculty staff were largely looking to minimise the number of disciplines participating in IPL activities, or to limit curriculum time. Some suggested extra curricula social activities to achieve IPL.

*So we may have to look at how we facilitate the social interaction of these [different discipline] groups because, let’s face it, they will learn a lot more from just sitting together, having a coffee and chatting*. (Faculty 2a)

Participants also offered different perspectives on the optimal timing of IPL experiences for students. The potential value of offering these experiences early in student learning before disciplinary identities are ingrained was noted.

*I guess that the particular thing that really grabbed my interest in this project was the fact that it was getting them [students] while they’re young I suppose. It’s before the roles have had a chance to be emblazed upon the various professions.* (Faculty 4c)

Participants spoke of the expectation that as educators and clinical supervisors they would provide IPL experiences to their students. However, in addition to the previously described lack of a clear definition of IPL, educational planning was hampered by the lack of consensus on how best to deliver IPL to achieve desired student learning outcomes, let alone what these outcomes might be. Once again there was an understanding that IPL was to be implemented, yet participants could not agree on how this could be done.

### The leadership hot potato

Activity theory also considers the distribution of labour within an activity system, that is who is responsible for what tasks to achieve the object. In relation to who would do what, participants were supportive in theory towards embedding IPL into core curriculum for students. However, they were also careful to stress the importance of their individual discipline roles and identities. One aspect of implementing IPL that received considerable attention related to the overall vulnerability of IPL activities when competing for resources within a core curriculum.

*So there’s a double jeopardy on the program. It’s [IPL] vulnerable, because it’s sitting outside the core curriculum and it’s vulnerable because the people who are advocating for it have, by virtue of the fact that they’re in this space and not being supported, they’re advocating outside the core curriculum too. So it’s set up to fail. It is doomed.* (Faculty 5)

Among the faculty participants there was considerable focus on the risks perceived to be attached to implementing IPL. These risks included tokenistic engagement.

*I think in this whole area there’s a risk of tokenism as well, which can be very counterproductive because people end up - it ends up reinforcing stereotypes rather than actually correcting stereotypes.* (Faculty 6b)

In relation to questions of who should lead a process of mainstreaming IPL, more junior participants felt senior staff should lead, while senior participants indicated that local champions (usually junior staff) should lead.

*IPL is one of those things that really has to be bottom up I think for it to work. If it’s top down then the suspicions are amplified. If it’s bottom up and it’s actually - you know if you have those few champions who can get the enthusiasm going I think then you can really build something really worthwhile.* (Faculty 4c)

*So I think a real key change has to come from top down leadership that says this is important. This is part of our curriculum and you are going to do this, rather than asking for expressions of interest. ...firmer top down direction as to what the job is and what the teaching load is. Otherwise, the faithful few who have passion will just get burnt out. ….. if the deans buy into it, then the deans enforce it and the deans make it happen.* (Faculty 5)

Implementation of IPL requires curriculum change champions. Despite general support for the idea of IPL, through their comments, a number of participants demonstrated little personal commitment to actual implementation of IPL in their own teaching practices. The general sense gained was one of paralysis in relation to grasping opportunities and leading change to achieve authentic IPL. The deans preferred ‘grass roots’ change whereas junior staff looked to their seniors to ‘mandate’ change. This contradiction in relation to distribution of tasks risks a lack of clearly identified champions with sufficient capacity and support to influence and effect change.

### Big expectations of IPL

Tensions were also evident in relation to the different perceptions of the value of IPL and by implication, which part of the curriculum were best suited to IPL activities. For some the value of IPL was expressed in terms of clinical practice outcomes many years post-graduation. A few participants spoke passionately to the belief that IPL was a means to address current health service delivery challenges. Others described a vision whereby IPL in particular, as distinct to other elements of the curriculum, was a vehicle for ensuring the ‘work-readiness’ of graduates.

*So my concept of inter-professional learning is about really changing the way we think about the work readiness [of graduates], the product, the outcome and think about how we create the mix of learning experiences using the expert disciplinarians to embed the concept of what it is to be a first-class nurse or a first-class doctor or a first-class dentist.* (Faculty 2a)

Some participants were more measured in their expectations of IPL and acknowledged that at times the benefits in terms of preparing a future health workforce may have been over emphasised. A small number of participants also noted that interprofessional collaboration in health care practice required complex skills and questioned whether these skills were teachable to students. The lack of a secure place within the core curriculum of health profession education programs was lamented and the ongoing mode of delivery of many IPL activities as a ‘bolt on’ curriculum structure perpetuated a vulnerability to misinterpretation and overstatement of benefits. For the passionate champions, IPL was associated with potential future benefits. However, more immediate and tangible benefits were illusive creating an ideal environment for diverse interpretations and unrealistic expectations to flourish.

### Resisting cultural change

The final contradiction identified related to the perceived mismatch between student experiences of IPL during their education, and their subsequent experiences as health practitioners in clinical practice. Throughout the interviews there were frequent references made to cultural factors being important influences on the uptake of IPL within the curriculum. Participants drew attention to the perceived cultural barriers to more efficient IPL. A commonly cited example was the lack of a supportive health service environment. Several participants made the further argument that until health systems and modes of service delivery change, IPL curriculum reform will flounder.

*People guard their territory and they’ve always done it this way. Why should we change? So there’s still quite a bit of that cultural stuff that needs to be shifted. I also have to say that the working culture within the health service doesn’t really support what we might be trying to do for our students.* (Faculty 2a)

Senior faculty staff were warier of IPL than were their more junior colleagues with particular concerns that discipline learning may be compromised. The resistance of staff to change was also discussed with comments suggesting tacit approval for colleagues to opt-out of IPL activities.

*There are going to be staff within our organisation that you’re probably never going to change and you have to minimise any negative role models that are in there.* (Faculty 1)

Although the need to tackle cultural challenges to increase uptake of IPL in the core curriculum was acknowledged by all participants, the influence of the hidden curriculum [35] and prevailing cultural norms was significant.

*I think also universities should not be thinking about this as a one-shot inoculation. I think for me one of the frustrations is that there’s only so much we can influence. Students do see practicum as the real curriculum, and that’s actually what matters. So they go into workplaces where they may or may not see inter-professional collaboration and we can’t - they might see poor behaviours and practices and we can’t influence that*. (Community health practitioner 9)

As has been previously described, there was a general lack of any sense of commitment or agency to achieving cultural change.

### The expansive learning cycle

We identified five different clusters of contradictions and then mapped these to the different stages of the ELC (Table 1). When considered together, these contradictions and tensions are likely to have an additive effect and impact. A lack of a definition that can be easily operationalised into, for example, specific learning outcomes, together with a lack of clarity around appropriate pedagogical approaches, provides an ideal environment for confusion in curriculum development and fragmented action. When the definition of IPL is unclear teachers may have concerns that disciplinary content will be compromised (ELC stage 2. analysis/double bind). A lack of agreement on learning and teaching activities among curriculum developers may undermine the confidence of students and teachers that learning will be effective (ELC stage 1. questioning/need state). It is hard to champion something that has unclear objectives. This inherent fragility is compounded if there is ongoing debate around leadership. For smaller disciplines, a lack of effective leadership models may raise concerns about inequitable discipline relations and representation in curriculum (ELC stage 1. questioning/need state). Without effective leadership, it is unclear who makes key decisions and importantly, who is responsible and accountable for delivery of IPL within the curriculum. In this environment, it is not surprising that some unrealistic expectations can take hold and flourish with multiple understandings of what IPL activities can be expected to achieve. When unrealistic expectations have developed around IPL, it would be an entirely reasonable concern among those responsible for curriculum development and delivery that their IPL curriculum will fall short on delivering on these expectations (ELC stage 2. analysis/double bind). Similarly, a lack of clarity around purpose or clear and committed leadership contributes to the ongoing status of IPL as an optional add-on to the core curriculum. Finally, if teachers believe that health services are not prepared for graduates skilled in IPL, teachers will fear their students will rapidly become disillusioned graduates (ELC stage 1. questioning/need state).

## Discussion

We entered into this study expecting to hear about the commonly cited challenges associated with implementing IPL such as timetabling constraints, cost, risk-averse departmental cultures and specific requirements set by individual disciplines and/or professional bodies [9–16]. The limited elaboration of possible solutions to these well described factors was somewhat unexpected. The participants expressed genuine concern that, among other things, the identities of individual disciplines were at risk if a more IPL based approach were to be adopted. If individuals are responding to a perceived risk of losing their disciplinary identity with protective strategies that unintentionally maintain the status quo, the result may be reinforcement of the importance of disciplinary learning. Identifying the underlying fears of, for example, loss of disciplinary identity, could facilitate and enable a more honest examination of factors hampering IPL implementation. Rather than challenging the protective behaviors directly then, it may be more effective to challenge the underlying fears and assumptions driving these behaviours [36]. Asking ‘what we are afraid of?’ might be highly informative. The recent identification of a set of IPL competencies that are equally applicable to all healthcare disciplines may assist this process by providing common ground for more flexible discussions [37].

Based on our analysis of the interviews from the perspective of activity theory, at least in the local environment, the identified contradictions had not yet generated sufficient tension to trigger an ELC. It seemed that participants were ‘stuck’ at the first or second stage (questioning and analysis) where they were still asking the ‘why’ and ‘what’ questions. They were resisting any movement into a space where they were ready to ‘break though’ and contemplate and then model new solutions. With an explicit focus on identifying and challenging underlying fears and assumptions in relation to IPL perhaps these identified contradictions should be highlighted to increase tensions and create an energy and momentum for change. When supported by an environment where innovative thinking and new solutions are entertained and supported, an ELC may be successfully completed [33].

While activity theory has been used in considerations of team work in clinical settings [32, 33, 38–41] its use in IPL is relatively novel. Activity theory has been applied to communication [42], student clinical placements [43], and team debriefings [44], but not specifically to curriculum development and implementation. Our study has used activity theory to reveal contradictions and tensions in IPL curriculum development and delivery.

As with many studies of this kind, the participants were drawn from one community. Although great care was taken to ensure the quality and credibility of the analysis, application of specific findings to other contexts and settings may require care. Further opportunities to test the findings of this study should be sought. To facilitate participation videoconference and telephone interviews were offered as an alternative to in person face to face interviews. While comparisons of face to face and telephone interview data show variable results in relation to any compromise to quality [45–47], it is likely that there was an impact on the volume of data obtained with the shorter telephone interviews.

The authors have conducted many previous studies of this type with data obtained through interview. This study differed from our usual experiences in two ways. Firstly, although participants were invited individually, several asked if they could bring another colleague to the interview, and three participants asked to be interviewed together. In some instances, the rationale was a modelling of interprofessional collaboration. In other instances, no reason was given. These requests were met. In doing so we acknowledge that this may have reduced our ability to access individual voices equally and to be confident that the views expressed where held by all participants. Participants may also have been more guarded in expressing opinions, especially if these opinions were felt to be more controversial. As it was beyond the scope of the approved study to explore why some participants made this request, we can only speculate on possible explanations. For example, some participants may have lacked confidence in their own knowledge of the area and preferred to be interviewed with others.

Secondly, we encountered great difficulty in exploring the challenges and barriers to implementing IPL in any great depth with participants and could not elicit any additional clarity on the problems to that which was already well articulated in the literature. The length of the interviews was considerably shorter than is usual for semi-structured interviews. Even with the use of prompts, participants moved quickly through the interview guide. Face to face interviews tended to be longer than telephone or videoconference interviews, and interviews with more than one participant were also longer in each case providing more data. We might speculate that as we were asking for information that was subsequently linked to underlying tensions, contradictions and possible perceived risks, some participants may have felt less comfortable to speak at greater length. It is plausible that this might have been a factor in some participants opting to be interviewed by telephone.

## Conclusion

Lack of agreement on optimal learning activities creates an unstable basis for curriculum development, delivery or evaluation. Unrealistic expectations can develop when there are multiple understandings of what IPL activities are expected to achieve. Within the theoretical perspective of activity theory, it can be argued that the most troublesome challenges in relation to implementing IPL could be embraced as contradictions that may lead to change. Seemingly intractable problems could be actively sought out and worked on to identify and address underlying fears and assumptions. The aim of this approach would be to generate the necessary tension to trigger an ELC and to promote new thinking. Importantly, novel thinking about timetabling, interdisciplinary relationships, leadership and governance should be encouraged. In reframing challenges as opportunities, the power of contradictions as levers for effective change might be more successfully accessed.

## Abbreviations

ELC: Expansive learning cycle
IPL: Interprofessional learning
